# A Case of Ectopic Pregnancy With an Unusually High Level of β-hCG Correlating With Normal Pregnancy

**DOI:** 10.7759/cureus.97989

**Published:** 2025-11-27

**Authors:** Fatimah A Al-Rufaye, Bahei Desouki

**Affiliations:** 1 Obstetrics and Gynaecology, Dorset County Hospital, Dorchester, GBR

**Keywords:** b-hcg, high level of b-hcg in ectopic pregnancy, ruptured ectopic pregnancy, ruptured tubal ectopic pregnancy, sonography in the diagnosis of ectopic pregnancy

## Abstract

This case report demonstrates an uncommon presentation of ectopic pregnancy. Diagnosis is usually based on clinical findings, ultrasound imaging, and serial serum beta-human chorionic gonadotropin (β-hCG) levels. However, β-hCG levels alone cannot reliably determine the location of a pregnancy, and in some cases, values may mimic those of a viable intrauterine pregnancy. This case highlights the diagnostic challenges when clinical symptoms suggest ectopic pregnancy despite β-hCG levels appearing consistent with normal intrauterine gestation.

We report the case of a 34-year-old woman at around six weeks of gestation who presented with sudden-onset right iliac fossa (RIF) pain. She denied vaginal bleeding and had not yet undergone an antenatal scan. Her past obstetric history patient includes one spontaneous miscarriage four months prior, with no other significant medical conditions. Her vital signs were normal and initial blood tests showed normal inflammatory markers and stable haemoglobin level. Serum β-hCG was 29,457 IU/L, which was correlating with her gestational age. Because of misleadingly high β-hCG levels and limited ultrasound availability over the weekend, other possible diagnoses, such as acute appendicitis or ruptured ovarian cyst, were initially considered. The patient underwent a transvaginal ultrasound (TVUSS) two days after admission, which confirmed an empty uterus and a left adnexal ectopic pregnancy with a fetal pole.

This case demonstrates a challenging presentation of ectopic pregnancy. Therefore, clinicians must maintain a high index of suspicion for ectopic pregnancy, even when β-hCG level resemble normal ranges for intrauterine pregnancy. Early imaging review is critical to avoid diagnostic delay and improve maternal outcomes.

## Introduction

Background: Ectopic pregnancy is defined as implantation outside the endometrial cavity, affects approximately 11 per 1,000 pregnancies in the UK, with around 12,000 diagnoses per year [1]. 95% of ectopic pregnancies are in the tube and rarely in the cervix, caesarean scar, ovaries and abdominal cavity [2,3]. Approximately 50% of patients with ectopic pregnancy have at least one identifiable risk factor, such as a previous ectopic pregnancy, use of an intrauterine contraceptive device at the time of conception, history of pelvic inflammatory disease, or smoking [3-5]. Clinical presentation can range from asymptomatic haemodynamically stable to intra-abdominal haemorrhage. However, the classical presentation is unilateral pelvic pain, bleeding, and positive pregnancy test [1,6].

Ectopic pregnancy is usually diagnosed in the first trimester of the pregnancy, and the most common gestational age at diagnosis is 6 to12 weeks [7,8]. Diagnosis of ectopic pregnancy typically involves clinical examination, biochemical testing and imaging [9]. Physical exam findings may include adnexal tenderness, cervical motion tenderness, or a palpable adnexal mass [10]. Beta-human chorionic gonadotropin (β-hCG) is an important diagnostic marker for ectopic pregnancy. The absence of an intrauterine gestation when β-hCG exceeds a threshold of 3,500 mIU/mL is suggestive of an abnormal pregnancy [3,11]. Studies on ectopic pregnancies have shown that β-hCG may not rise as expected and can remain lower than in normal pregnancies [3,8,11-13]. Imaging involves transvaginal ultrasound (TVUSS), which helps diagnose the majority of women with ectopic pregnancy. Diagnosis by ultrasound should be based on the identification of an adnexal mass rather than an empty uterus [14].

Although the mortality rates have declined, ectopic pregnancies remain the leading cause of maternal mortality in early pregnancy. Therefore, early and quick diagnosis of ectopic pregnancy is essential to prevent potential complications [15].

We report this case, which demonstrates an unusual presentation of ectopic pregnancy with a markedly elevated β-hCG level, to emphasize the importance of maintaining a high clinical index of suspicion in order to avoid delayed diagnosis and improve patient outcomes.

## Case presentation

We report a case of a 34-year-old lady who was approximately six weeks pregnant and presented to the emergency department on a Saturday evening with sudden central abdominal pain that later migrated to her right iliac fossa (RIF), associated with nausea and vomiting. She denied vaginal bleeding, shoulder tip pain, and urinary or bowel problems, and had not yet undergone an antenatal scan. Her past obstetric history included one spontaneous miscarriage 4 months prior, otherwise, no other medical conditions.

Clinical examination revealed marked tenderness in both the RIF and suprapubic regions without signs of local peritonitis. Vaginal examination revealed no bleeding or cervical excitation. She was haemodynamically stable apart from postural hypotension, and initial blood tests on admission showed normal inflammatory markers and haemoglobin level of 129 g/L. Serum β-hCG was 29,457 IU/L (Table 1). These findings led clinicians to consider alternative diagnoses, including acute appendicitis, ruptured ovarian cyst, and corpus luteum haemorrhage.

**Table 1:** Blood investigations β-hCG: Beta-human chorionic gonadotropin; CRP: C-reactive protein; HB: Haemoglobin; WCC: White cell count

Parameters	On admission (16.04 p.m.)	Day 1 (6.13 a.m.)	Day 1 (12.57 p.m.)	Post op (20.33 p.m.)	On discharge	2 weeks post op	Normal ranges
HB (g/L)	129	107	114	108	100	-	120-150
β-hCG (IU/L)	29457	24796	-	-	-	292	0-5
WCC (x 10^9^/L)	14.3	8.5	8	10.7	9.4	-	4-10
CRP (mg/L)	1.7	3.7	5	3	11	-	<5

The general surgical team evaluated the patient for suspected acute appendicitis and advised ruling out ectopic pregnancy, given the normal inflammatory markers. The patient was admitted under joint care by the gynaecology and general surgical teams.

During her stay, she remained haemodynamically stable. However, her haemoglobin dropped from 129 to 107 g/L within six hours (Table 1). She also had a repeat β-hCG at 12 hours post admission, which came back at 24,796 IU/L (Table 1). She continued to complain from RIF pain despite regular analgesia.

Because of limited TVUSS availability over the weekend, the patient initially underwent a transabdominal ultrasound, which demonstrated a normal uterus, no adnexal masses, and mildly increased fluid in the pouch of Douglas (POD). She remained haemodynamically stable, with no signs of peritonitis and stable haemoglobin levels (Table 1). The patient was counselled regarding the available options for excluding ectopic pregnancy and acute appendicitis. However, she was reluctant to undergo a diagnostic laparoscopy without a confirmed diagnosis due to concerns about the potential risks of general anaesthesia to the pregnancy. This complex situation prompted the clinical team to consider delaying intervention and awaiting transvaginal ultrasonography to help exclude ectopic pregnancy, given her haemodynamic stability.

On Monday morning, a TVUSS scan confirmed a left tubal ectopic pregnancy measuring 4.3 x 3.4 x 4.2 cm containing a gestational sac and foetal pole with no cardiac activity and with a large amount of blood in the POD. The patient remained haemodynamically stable with stable haemoglobin level (Table 1).

**Figure 1:**
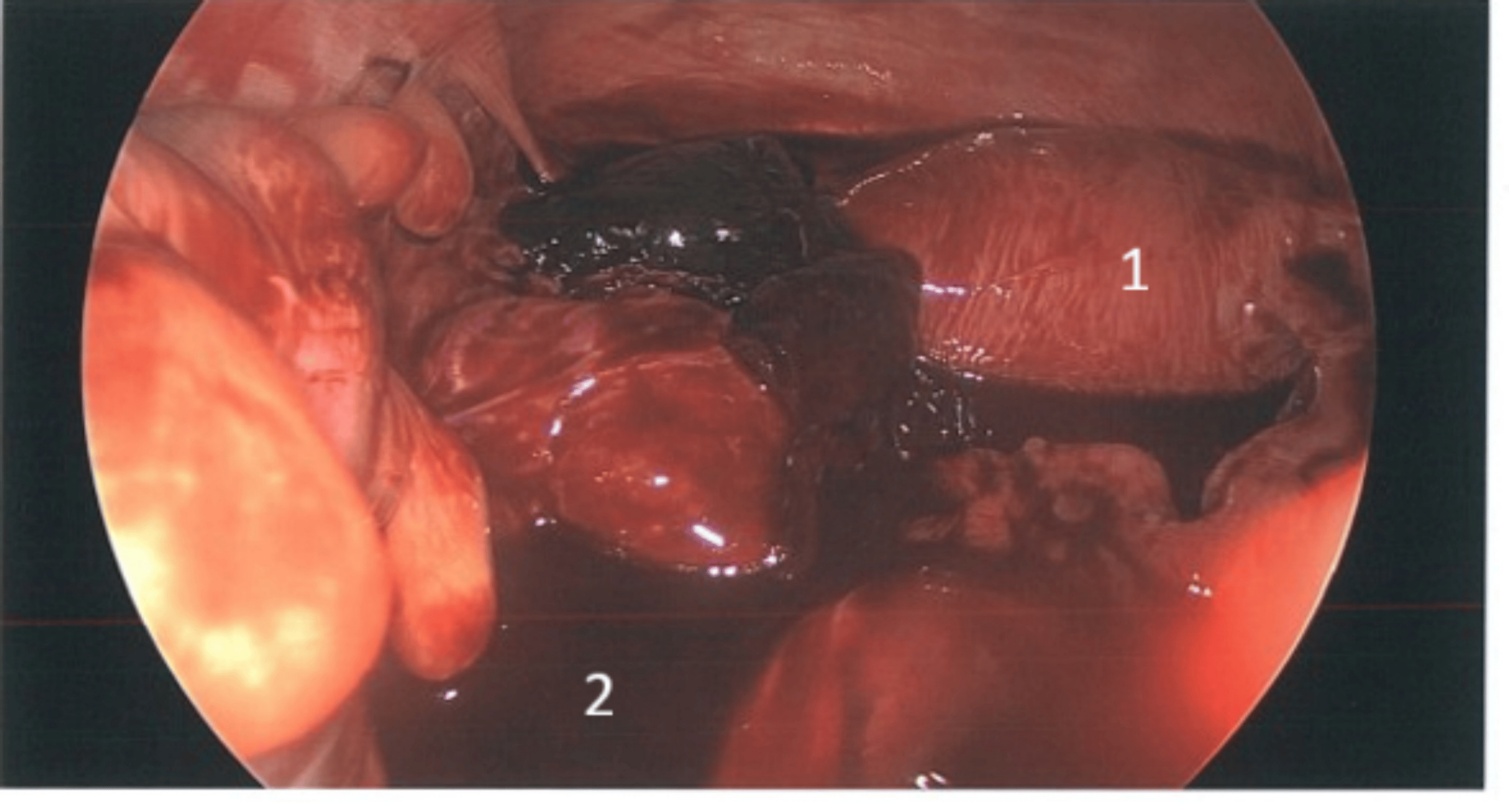
Rupture ectopic pregnancy 1: Uterus; 2: Hemoperitoneum

**Figure 2:**
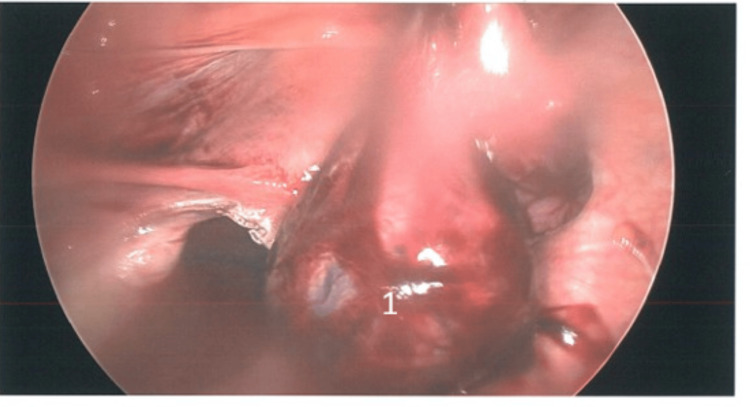
Hemoperitoneum and left tubal ectopic pregnancy 1: Left ruptured tubal ectopic pregnancy

**Figure 3:**
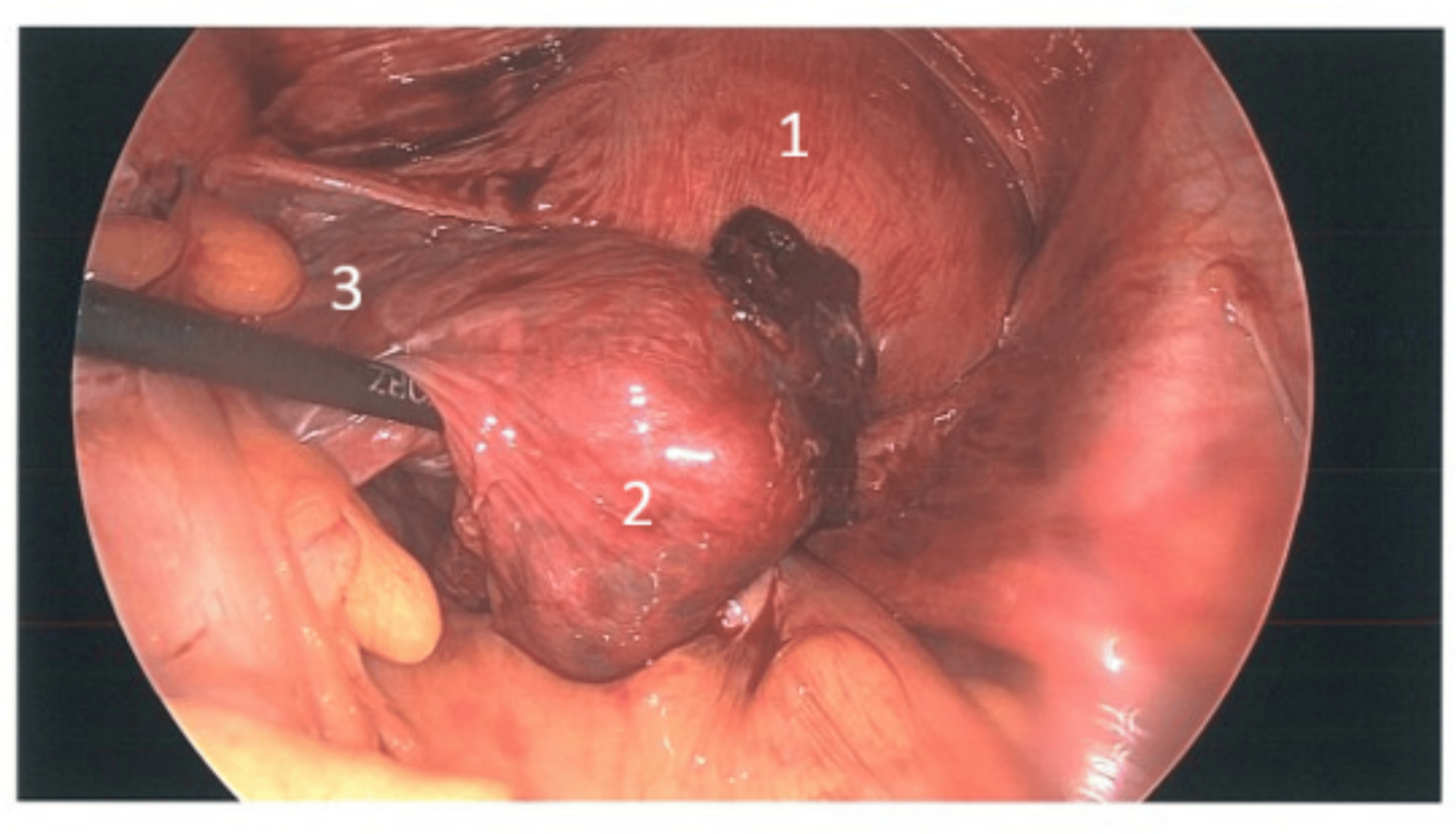
Left tubal ectopic pregnancy 1: Uterus; 2: Left tubal ectopic pregnancy; 3: Left tube

The patient was taken to the operating theatre the same day and underwent laparoscopic surgery. Intra-operatively, a ruptured left tubal ectopic pregnancy was confirmed (Figures 1,2,3), with about 500 mL of hemoperitoneum. A left salpingectomy was then performed. The procedure was uneventful, and she made a satisfactory recovery and was discharged the next day with stable haemoglobin level (Table 1). Follow-up β-hCG measured 292 IU/L (Table 1), and histopathology confirmed ectopic pregnancy.

## Discussion

This case demonstrated a ruptured tubal ectopic pregnancy despite β-hCG level that appeared consistent with a normal intrauterine pregnancy, an uncommon and diagnostically challenging presentation [16]. However, several studies have shown that a single β-hCG level does not indicate the location of the pregnancy; only serial β-hCG can help confirm the viability of pregnancy rather than the location of it [8,17]. Studies also indicate that as many as 20.8% of ectopic pregnancies can exhibit β-hCG rise patterns similar to those seen in normal pregnancies [17-19]. Therefore, understanding the role of β-hCG in the diagnosis of ectopic pregnancy is vital for early detection. Close monitoring of β-hCG with other diagnostic tools can help provide prompt and effective care.

This case also demonstrated the limitations of transabdominal ultrasound in diagnosing ectopic pregnancy, as it may mislead clinicians to consider alternative possibilities. Studies have shown that the diagnosis of ectopic pregnancy should be based on the presence of an adnexal mass rather than an empty uterus [14]. They also indicate that TVUSS is the new gold standard and the diagnostic tool of choice for all forms of ectopic pregnancy, including both tubal and non-tubal types [14]. The vast majority of women presenting with an ectopic pregnancy can be reliably diagnosed using TVUSS as a single, stand-alone test [14].

Looking back at how this difficult case was managed, the very high β-hCG level made the diagnosis less clear and led clinicians to consider other possible conditions. The patient’s reluctance to have a diagnostic laparoscopy without a confirmed diagnosis also made the situation more challenging. These factors together caused a delay in diagnosis and resulted in more blood loss during surgery, even though she was regularly monitored, had frequent blood tests, and was reviewed by the appropriate teams.

Considering all of the above the key learning points from this case will be that a single β-hCG level cannot determine the location of a pregnancy, as even high levels may be seen in ectopic pregnancies. Serial β-hCG measurements and early TVUSS are essential for accurate diagnosis. It also shows how atypical findings and patient reluctance for invasive procedures can delay diagnosis and increase risk. Early use of appropriate imaging, good clinical judgement, and clear communication with patients are key to preventing complications.

## Conclusions

This case highlights that β-hCG levels alone cannot determine the location of a pregnancy and may occasionally mimic normal levels in cases of ectopic pregnancy. Therefore, diagnostic imaging should be performed in any patient with clinical suspicion of ectopic pregnancy, regardless of β-hCG concentration. Clinicians should maintain a high index of suspicion, particularly when clinical symptoms are inconsistent with β-hCG trends. Timely imaging assessment and close follow-up are essential to prevent diagnostic delays and improve maternal outcomes.
